# Revision of the genus *Salganea* Stål (Blattodea, Blaberidae, Panesthiinae) from China, with descriptions of three new species

**DOI:** 10.3897/zookeys.412.7134

**Published:** 2014-05-29

**Authors:** Xiudan Wang, Yan Shi, Zongqing Wang, Yanli Che

**Affiliations:** 1 Institute of Entomology, College of Plant Protection, Southwest University, Beibei, Chongqing 400716, China

**Keywords:** New species, new synonym, new combination, cockroaches, *Panesthia*

## Abstract

Three new species of *Salganea* Stål, 1877 are described and illustrated: *S. quinquedentata* **sp. n.**, *S. anisodonta* **sp. n.** and *S. flexibilis* **sp. n.** *S. taiwanensis* Roth, 1979, *S. guangxiensis* (Feng & Woo, 1990), *S. incerta* (Brunner von Wattenwyl, 1893) and *S. raggei* Roth, 1979 are redescribed. *Panesthia concinna* Feng & Woo, 1990 is synonymized with *S. taiwanensis* Roth, 1979 and *Panesthia guangxiensis* Feng & Woo, 1990 is transferred to the genus *Salganea* for the first time. As well, a key to species from China is presented.

## Introduction

The blaberid genus *Salganea* belongs to the subfamily Panesthiinae (tribe Salganeini), which is subsocial and xylophagous. Some members live in biparental families (Maekawa et al. 2005), where their young nymphs are defended and fed by parents (Maekawa et al. 2008). They are heavy bodied insects ranging from 15.5 to >60 mm in length and the pronotum of the two sexes is usually similar (Maekawa et al. 2008), which can result in a substantial ecological impact on the decomposition of logs (Bell et al. 2007).

This genus is recognized by its *T6* with lateral margin even, *T7* with lateral margin serrated, and holes associated with setae in the anterolateral corners of abdominal terga. Princis (1965) listed 22 species of *Salganea* worldwide. Bey-Bienko (1957) listed 3 species of *Salganea* from Sichuan and Yunnan Provinces, China, 2 of which, *Salganea amboinica* (Brunner von Wattenwyl, 1893) and *Salganea morio* (Burmeister, 1838), probably referred to *Salganea taiwanensis* and *Salganea raggei* respectively (Roth 1979). Roth (1979) reported 42 species and 4 subspecies in this genus, 3 species of which were from China. In addition, he erected 5 species-groups, based on the anterior margin of the pronotum and male genital phallomere *L2d*, *i.e.*, the *papua* species-group, the *foveolate* species-group, the *raggei* species-group, the *morio* species-group and the *nigrita* species-group, although the molecular analysis result did not conform to this conclusion completely (Maekawa et al. 2001). Asahina (1988) considered that *Salganea taiwanensis* was composed of two subspecies on the basis of the body size, male genital phallomere *L2d* and on distribution as well. But according to the molecular phylogeny and geographic distribution of wood-feeding cockroach genera *Salganea* Stål, 1877 and *Panesthia* Serville, 1831 in East Asia Islands, Maekawa et al. (1999a) did not agree with the subspecies designations of Asahina. Maekawa et al. (1999b) described one species and then described one species, providing the phylogenetic tree for 27 species and 3 subspecies of the genus *Salganea* in 2005. Up to now, there are 47 species and 6 subspecies recognized worldwide (Beccaloni 2007), of which 5 species are from China.

Herein, we redescribe *Salganea*, describe 3 new species from China, redescribe 4 species and give a key to the Chinese species. After the examination of type specimens, *Panesthia concinna* Feng & Woo, 1990 is synonymized with *Salganea taiwanensis* and *Panesthia guangxiensis* Feng & Woo, 1990 is transferred to the genus *Salganea*.

## Materials and methods

The terminology of the head, body and male genitalia used in this paper mainly follows Roth (1977, 1979), and the terminology of veins follows Rehn (1951). Measurements were based on material examined, and the measurement of body length was without the tegmen. CV_total_ is the coefficient of variation of total number of veins which without radius and anal veins (Liang et al. 2012). The genital segments of the examined specimens were macerated in 10% NaOH and observed in glycerin jelly using a Motic K400 stereomicroscope. All drawings were made with the aid of a Motic K400 stereomicroscope. Photographs of the specimens were made using a Canon 50D plus a Canon EF 100mm f/2.8L IS USM Macro lens with the aid of the Helicon Focus software. The type specimens are deposited in the Insect Collection of Southwest University, Beibei, Chongqing, China (SWU) and the Museum of Hebei University, Baoding, Hebei Province, China (HBU).

## Taxonomy

### Family Blaberidae Brunner von Wattenwyl, 1865
Subfamily Panesthiinae Kirby, 1904

#### Genus Salganea

Stål, 1877

http://species-id.net/wiki/Salganea

Salganea Stål, 1877: 37. Type species: *Panesthia morio* Burmeister, 1838; Roth 1979: 4; Asahina 1988: 257.
Mylacrina Kirby, 1903: 414. Type species: *Mylacrina wrayi* Kirby, 1903. Synonymized by Roth 1977: 60.

##### Generic diagnosis

**(modified after Roth (1979)).** Vertex rarely foveolate (except *Salganea papua* and *Salganea mandelsi*) and exposed. Pronotum transversal ovate, anterior margin almost straight or slightly convex, lateral margins arched, hind margin almost straight; anterior half depressed, with a pair of curved, oblique grooves; posterior half elevated and punctate. Tegmina and wings fully developed, sometimes mutilated, or reduced, or tegmina reduced but wings absent, or both tegmina and wings absent. If tegmina and wings fully developed, tegmina usually narrow and leathery with base thickened, the humeral area well developed and punctate, anterior margin slightly curved medially, subcostal vein short and unbranched. Hind wings with anal area developed with border smooth and rounded; subcostal vein (*Sc*) straight and simple or with a small branch at apical part, extending beyond the midline of wing length; radial vein (*R*) with less than five branches, with or without apical posterior branch (*Ap. Post. Br.*); median vein (*M*) simple, rarely with a terminal twigging; cubitus (*Cu*) with more than 8 branches, at least half of which are incomplete and fuse apically or going to the first plical vein (1*Pl*). Lateral margin of the 6^th^ abdominal tergum (*T6*) straight; lateral margin of the 7^th^ abdominal tergum (*T7*) uneven, caudal angles produced caudally or laterocaudally; anterolateral corners of 6^th^ and 7^th^ abdominal terga usually with holes, sometimes holes also existing in the corners of *T3*, *T4* or *T5*, which are usually accompanied with small setae or hairs. Abdominal sternites punctured, the last sternite with a marginal ridge extending along the lateral margins. Supra-anal plate transverse; paraprocts broad, left one in ventral view armed with a finger-like projection, which is curved dorsally and whose apex is sclerotized. Subgenital plate flabellate, anterior margin straight or concave, lateral margins oblique, hind margin round. Four genital phallomeres as below: first sclerite of left phallomere (*L1*) plate-like, well developed but usually not sclerotized, sometimes reduced or absent; second ventromedial sclerite of left phallomere (*L2vm*) rod-like; second dorsal sclerite of the left phallomere (*L2d*) elongate, variable; second sclerite of right phallomere (*R2*) hook-shaped, reduced or absent in some species.

##### Remarks.

Because the irregularities on lateral margin of *T7* maybe subobsolete, this genus is similar to *Panesthia*. With a notch existing anterior to the laterocaudal angle of *T7* sometimes, this genus resembles *Ancaudellia* Shaw, 1925. But it can be distinguished by the following characteristics: 1) anterolateral corners of 6^th^ and 7^th^ abdominal terga usually with holes and holes associated with well spaced setae (*Panesthia* rarely with holes and without setae, *Ancaudellia* with grooves and associated with dense patches of contiguous setae); 2) lateral margin of *S7* with a ridge extending from anterior margin to hind margin (with a feeble and short ridge, or without a ridge in *Panesthia* and *Ancaudellia*).

Although *Salganea amboinica*, *Salganea morio* and *Salganea passaloides passaloides* (Walker, 1868) were recorded from China, none of them were found after our wide survey of collecting and examining loans from other museums. Moreover, Roth (1979) had questioned whether they were truly recorded from China. These species have been excluded in our count for the total number of species in this genus from China, and have been removed from our key to the *Salganea* from China.

##### Distribution.

Few places of Palaearctic Region (China, Japan), Oriental Region, Australian Region.

##### Key to species of *Salganea* from China (males)

**Table:** 

1	*L2d* bifurcated (*nigrita* species-group)	2
–	*L2d* not bifurcated (*raggei* species-group)	8
2	Tegmina and wings reduced, not reaching apex of metanotum	3
–	Tegmina and wings fully developed, surpassing apex of abdomen	4
3	Anterior margin of pronotum with a V-shaped mesal excision and a small reflexed tubercle on each side of the indentation, anterolateral corners of *T5*–*T6* with holes and associated setae	*Salganea biglumis*
–	Anterior margin of pronotum hardly excised mesally or smooth, with a small unobvious tubercle on each side of the indentation, anterolateral corners of *T6*–*T7* with holes but lacking associated setae	*Salganea gressitti*
4	*R2* reduced, usually not hook-like or absent	5
–	*R2* developed, hook-like	6
5	Hind margin of supra-anal plate with 8-13 triangular teeth (Fig. 45)	*Salganea taiwanensis*
–	Hind margin of supra-anal plate with 10-11 obtuse rounded teeth (Fig. 22)	*Salganea guangxiensis* comb. n.
6	Hind margin of supra-anal plate with 9-13 contiguous triangular teeth (Fig. 55)	*Salganea incerta*
–	Hind margin of supra-anal plate with relatively spaced teeth	7
7	Pronotum with a pair of tubercles mesially (Fig. 33), hind margin of supra-anal plate with 7-8 stubby subacute teeth (Fig. 36)	*Salganea anisodonta* sp. n.
–	Pronotum without tubercles (Fig. 25), hind margin of supra-anal plate with 5 relatively slender teeth, sometimes fused together or with small acute spines between them (Fig. 28)	*Salganea quinquedentata* sp. n.
8	Lateral margin of *T7* with 5-6 obtuse teeth (Fig. 62), hind margin of supra-anal plate with 8-16 subequal teeth (Fig. 64)	*Salganea raggei*
–	Lateral margin of *T7* with 3 subacute teeth (Fig. 70), hind margin of supra-anal plate with 8 unequal teeth (Fig. 72)	*Salganea flexibilis* sp. n.

#### Salganea quinquedentata sp. n.

http://zoobank.org/863217EA-CB48-41C7-9AC0-980E1A0B775C

http://species-id.net/wiki/Salganea_quinquedentata

Figs 1–4 , 21–28 , 80–81 , 89–90

##### Description.

**Figures 1–20.**
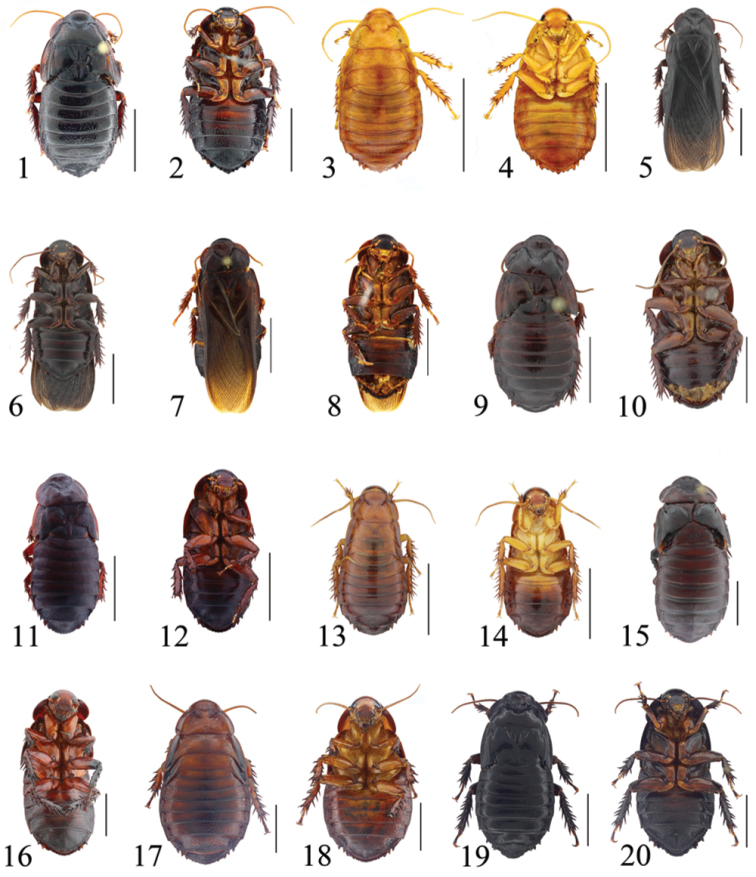
**1–2** *Salganea quinquedentata* sp. n., male: **1** holotype, dorsal view **2** same, ventral view **3–4** *Salganea quinquedentata* sp. n., nymph: **3** paratype, dorsal view **4** same, ventral view **5–6** *Salganea anisodonta* sp. n., male: **5** holotype, dorsal view **6** same, ventral view **7–8** *Salganea taiwanensis* Roth, 1979, male: **7** holotype of *Panesthia concinna* Feng & Woo, 1990, dorsal view **8** same, ventral view **9–10** *Salganea guangxiensis* (Feng & Woo, 1990), male: **9** holotype of *Panesthia guangxiensis* Feng & Woo, 1990, dorsal view **10** same, ventral view **11–12** *Salganea incerta* (Brunner von Wattenwyl, 1893), male: **11** dorsal view **12** ventral view **13–14** *Salganea incerta* (Brunner von Wattenwyl, 1893), nymph: **13** dorsal view **14** ventral view **15–16** *Salganea raggei* Roth, 1979, male: **15** dorsal view **16** ventral view **17–18** *Salganea raggei* Roth, 1979, nymph: **17** dorsal view **18** ventral view **19–20** *Salganea flexibilis* sp. n., male: **19** holotype, dorsal view **20** same, ventral view. Scale bars = 1.0 cm.

**Male.** Body dark reddish brown, darker or black on caudal segments (Fig. 1). Eyes brown and ocelli yellowish. Antennae, upper lip, mandible, labial palpi and maxillary palpomeres brown. Legs reddish brown with coxae and trochanter brown. Abdominal sternites reddish brown with the middle of anterior three sternites brown (Fig. 2).

**Figures 21–28.**
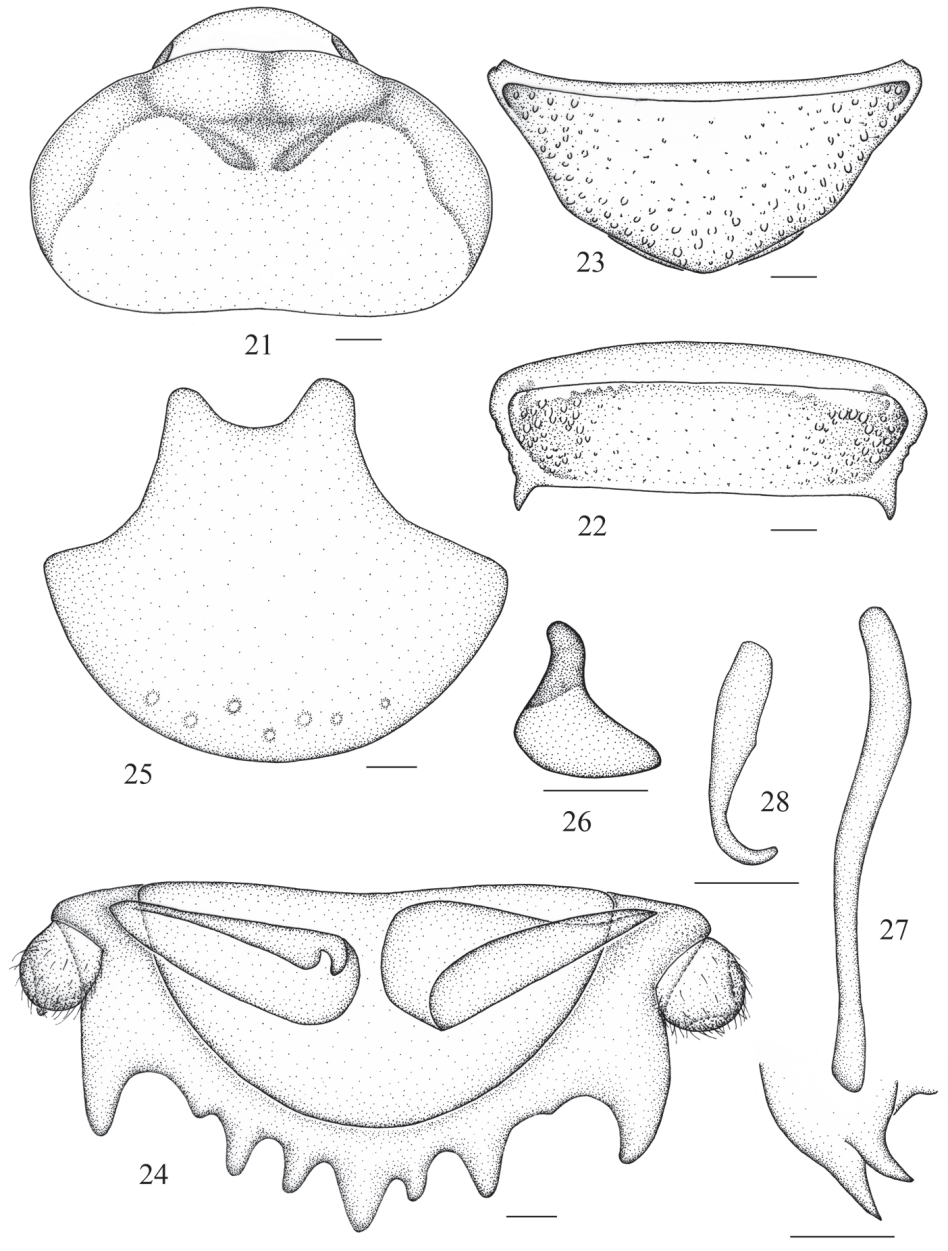
*Salganea quinquedentata* sp. n. **21** vertex and pronotum **22** abdominal tergum 7, dorsal view **23** abdominal sternite 7, ventral view **24** supra-anal plate and paraprocts, ventral view **25** subgenital plate, dorsal view **26** left phallomere (*L1*) **27** median phallomere (*L2vm* and *L2d*) **28** right phallomere (*R2*). Scale bars = 1.0 mm (Figs **21–23**), 0.5 mm (Figs **24–28**).

Vertex and face punctate, the former exposed. Anterior margin of pronotum smooth, or weakly concave; anterior half of pronotum slightly depressed, the floor punctured, denser laterally; posterior half punctured sparsely and almost evenly, without tubercles (Fig. 21). Tegmina and wings well developed, extending beyond end of abdomen, sometimes mutilated (Fig. 1). Radius of tegmen with a long apical posterior branch, which has accessory branches, or apical posterior branch absent; median vein is simple or branched (Figs 80–81). Radial vein of hind wing with posterior branch medially; median vein branched terminally or not; cubitus with 4–5 complete and 5–6 incomplete branches (Figs 89–90). Anterior ventral margin of front femur with 1–3 spines and a small distal spine, hind margin with a large distal spine. Abdominal tergites punctured, the punctures denser laterally and caudally; *T5*–*T7* with gradually increased holes on the anterolateral corners, minute sparse hairs sometimes visible on the surfaces; caudal angles of *T6* weakly explored; lateral margins of *T7* slightly uneven, caudal angles oblique, large and tapering (Fig. 22). Abdominal sternites densely punctured, the punctations larger and denser caudally; hind margin of the last sternite entire (Fig. 23). Supra-anal plate densely punctured, coarser than abdominal tergites; hind margin with 5 subacute and symmetrically slender teeth, which are deflexed and widely spaced, the largest one situated in the middle; teeth with margin smooth or small acute spines between the teeth, sometimes teeth fused together; lateral angles larger than the medial tooth. Cercus without setae dorsally, ventral surface convex with dense hairs (Fig. 24). Anterior margin of subgenital plate concave, anterolateral corners rounded; lateral margins concave (Fig. 25).

**Male genitalia.** Genital phallomere *L1* reduced, only a short lobe remaining, or absent (Fig. 26); *L2d* tapering at apex, with a relatively large lateral lobe (Fig. 27); *R2* weakly curved, hook-shaped (Fig. 28).

**Female.** Essentially similar to male, difficult to distinguish externally.

**Nymph.** Body yellowish brown and eyes dark. Hind margin of the supra-anal plate with 5 contiguous and triangular teeth, sometimes separated by tiny tines. Remaining external morphological features are characteristic of the adult (Figs 3–4).

##### Measurements.

Male, 3^th^–5^th^ maxillary segments: 0.57–0.67/0.48–0.87/0.61–1.00mm; pronotum: length × width: 5.2–5.5 × 8.8–9.3mm; tegmen: 24.1–25.0mm; body length: 26.9–29.5mm; fore leg: coxae: 2.29–2.51mm, trochanter: 1.46–1.79mm, femur: 3.24–3.85mm, tibia: 1.19–2.61mm, 1^st^–5^th^ tarsus: 0.57–0.69/0.20–0.24/0.19–0.24/0.28–0.32/1.00–1.28mm; mid leg: coxae: 2.48–2.90mm, trochanter: 2.33–2.58mm, femur: 5.01–5.17mm, tibia: 3.84–4.06mm, 1^st^–5^th^ tarsus: 1.03–1.12/0.25–0.28/0.24–0.27/0.33–0.35/1.00–1.24mm; hind leg: coxae: 2.14–2.53mm, trochanter: 2.70–2.75mm, femur: 5.23–5.81mm, tibia: 5.47–6.14mm, 1^st^–5^th^ tarsus: 1.18–1.22/0.27–0.29/0.27–0.31/0.27–0.30/1.07–1.15mm; cerci: 0.64–0.97mm.

Female, 3^th^–5^th^ maxillary segments: 0.73–0.79/0.84–0.85/0.93–1.08mm; pronotum: length × width: 5.0 × 8.8–9.5mm; body length: 26.5–27.5mm; fore leg: coxae: 2.17–2.29mm, trochanter: 0.61–2.30mm, femur: 1.94–2.07mm, tibia: 2.16–2.64mm, 1^st^–5^th^ tarsus: 0.63–0.80/0.24–0.28/0.23–0.28/0.32–0.27/0.91–1.20mm; mid leg: coxae: 2.81–2.89mm, trochanter: 2.55–2.87mm, femur: 5.31–5.76mm, tibia: 4.52–4.60mm, 1^st^–5^th^ tarsus: 1.22–1.23/0.28–0.30/0.25–0.27/0.32–0.34/1.06–1.45mm; hind leg: coxae: 2.06–2.28mm, trochanter: 2.53–2.92mm, femur: 5.00–6.23mm, tibia: 5.47–7.32mm, 1^st^–5^th^ tarsus: 1.36/0.35/0.29/0.36/1.39mm; cerci: 0.87–0.91mm.

##### Material examined.

*Holotype*, male, China: Hainan Prov., Lingshui County, Mt. Diaoluoshan, 18°43.462'N, 104°52.105'E, 4 May 2013, coll. Yan Shi and Shunhua Gui (SWU). *Paratypes*, two males, three females and six nymphs, same data as holotype (SWU); one female, Hainan Prov., Mt. Wuzhishan, 2 May 1964, coll. Yuliang Luo (SWU).

##### Remarks.

This species is assigned into the *Salganea nigrita* species group by the forked *L2d*. It resembles *Salganea incerta*, but can be distinguished by the following characteristics: 1) anterior margin of pronotum entire and without tubercles, indented and with tubercles in *Salganea incerta*; 2) the floor of pronotum without tubercles, with tubercles in *Salganea incerta*; 3) hind margin of seventh abdominal sternite entire, the latter with a medial excision; 4) hind margin of supra-anal plate with 5 distinct, subacute and slender teeth, with 9-13 triangular teeth in *Salganea incerta*.

##### Etymology.

The specific epithet is derived from the Latin word “*quinquedentatus*”, referring to the posterior margin of supra-anal plate with 5 distinct and slender teeth.

#### Salganea anisodonta sp. n.

http://zoobank.org/45FEEBFC-E670-492B-8FB2-6E9CB92C01B4

http://species-id.net/wiki/Salganea_anisodonta

Figs 5–6 , 29–36 , 82 , 91

##### Description.

**Male.** Body dark reddish brown (Fig. 5). Face black, eyes dark brown, ocelli yellowish, upper lip and mandible brown; antennae, labial palpi and maxillary palpomeres dark brown. Legs reddish brown with coxae and trochanter brown. Abdominal sternites reddish brown, darker caudally (Fig. 6).

**Figures 29–36.**
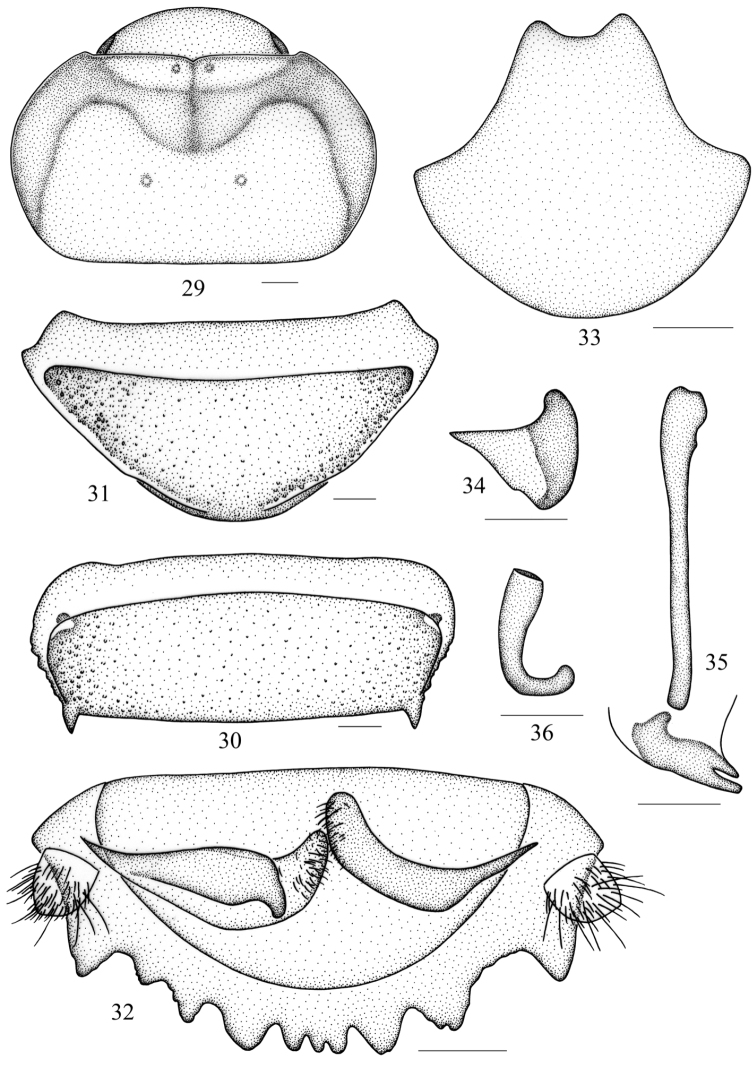
*Salganea anisodonta* sp. n. **29** vertex and pronotum **30** abdominal tergum 7, dorsal view **31** abdominal sternite 7, ventral view **32** supra-anal plate and paraprocts, ventral view **33** subgenital plate, dorsal view **34** left phallomere (*L1*) **35** median phallomere (*L2vm* and *L2d*) **36** right phallomere (*R2*). Scale bars = 1.0 mm (Figs **29–33**), 0.5 mm (Figs **34–36**).

Vertex exposed and without punctures. Face punctulated, ocelli round and with border distinct. Anterior margin of pronotum with a V-shaped excision mesially, a small recurved tubercle on the each side of the indentation; anterior 1/3 half of pronotum depressed, the floor densely granular; lateral and posterior half punctured, with a pair of small tubercles in the middle (Fig. 29). Tegmina and wings mutilated, probably well developed (Fig. 5). Radial vein of tegmen with a long apical posterior branch mesially, which is branched at apex and with an accessory branch; the median vein branched before the midline (Fig. 82). Hind wing with subcostal vein branched at apical part; radial vein bifurcated at apex and forked medially; median vein simple; cubitus with 6 complete and 5 incomplete branches (Fig. 91). Anterior ventral margin of front femur without spines, hind margin with a large distal spine. Abdominal tergites punctured, the punctures denser and larger laterally; *T7* with coarse surface scattered with sparse setae, and with circular depressions laterally, lateral margin crenulate, caudal angles oblique and tapering (Fig. 30). *T6*–*T7* with small holes on the anterolateral corners, which is associated with minute sparse hairs in the openings. Abdominal sternites densely punctured; hind margin of *S7* slightly convex and subgenital plate weakly exposed (Fig. 31). Supra-anal plate extremely coarse, hirsute and covered with depressions similar to *T7* in density; hind margin with 7-8 relatively separated teeth, which have an uneven border with lateral ones larger than teeth in the middle, or two fused together; lateral angles equal or larger than the biggest tooth between them. Cercus basiconic, with ventral side swollen and with setae ventrally and dorsally (Fig. 32). Anterior margin of subgenital plate slightly concave or more or less straight, lateral margin curved inwards (Fig. 33).

**Male genitalia.** Genital phallomere *L1* reduced to a small plate (Fig. 34); *L2d* tapering and bifurcated apically, major branch with apex rounded and a little larger than the lateral one (Fig. 35); *R2* developed and curved, hook-shaped, with apex obtuse and slightly swollen (Fig. 36).

**Female.** Anterior margin of pronotum with smaller tubercles than male and the tubercles not recurved.

**Nymph.** Unknown.

##### Measurements.

Male, 3^th^–5^th^ maxillary segments: 0.53–0.69/0.76–0.78/0.61–0.97mm; pronotum: length × width: 5.0–5.1 × 8.4–8.6mm; distance between disc tubercles: 1.9–2.1mm; tegmen: 28.5mm; body length: 27.5–28.0mm; fore leg: coxae: 2.63–2.83mm, trochanter: 1.50–1.97mm, femur: 3.24–3.76mm, tibia: 1.73–2.33mm, 1^st^–5^th^ tarsus: 0.73/0.35/0.26/0.34/1.30mm; mid leg: coxae: 2.70–2.93mm, trochanter: 2.19–2.72mm, femur: 4.54–4.85mm, tibia: 3.72–3.98mm, 1^st^–5^th^ tarsus: 1.02–1.16/0.26–0.27/0.24–0.28/0.29–0.34/1.02–1.22mm; hind leg: coxae: 2.69–2.74mm, trochanter: 2.55–2.65mm, femur: 5.13–5.38mm, tibia: 5.37–5.76mm, 1^st^–5^th^ tarsus: 1.24/0.28/0.27/0.32/1.10mm; cerci: 0.67–0.74mm.

Female, 3^th^–5^th^ maxillary segments: 0.60/0.82/0.92mm; pronotum: length × width: 5.6 × 9.1mm; distance between disc tubercles: 2.2mm; body length: 29.6mm; fore leg: coxae: 2.45mm, trochanter: 2.05mm, femur: 3.44mm, tibia: 2.42mm, 1^st^–5^th^ tarsus: 0.65/0.32/0.29/0.32/0.95mm; mid leg: coxae: 2.99mm, trochanter: 2.84mm, femur: 5.20mm, tibia: 3.90mm, 1^st^–5^th^ tarsus: 1.03/0.27/0.26/0.29/1.19mm; hind leg: coxae: 2.72mm, trochanter: 3.12mm, femur: 5.75mm, tibia: 6.30mm, 1^st^–5^th^ tarsus: 1.41/0.37/0.30/0.40/1.37mm; cerci: 0.66mm.

##### Material examined.

*Holotype*, male, China: Yunnan Prov., Longling County, Longxin Township, Heishan Village, 2300m, 23–25 December 2008, coll. Jishan Xu and Zhenhua Gao (HBU). *Paratypes*, one male and one female, same data as holotype (HBU).

##### Remarks.

This species is placed into the *Salganea nigrita* species group and is similar to *Salganea incerta*, but can be distinguished by: 1) anterior margin of pronotum with tubercles in female, without or weakly indicated in female of *Salganea incerta*; 2) anterior margin with two reflexed tubercles mesially, tubercles in *Salganea incerta* not reflexed; 3) hind margin of supra-anal plate with 7-8 relatively separated teeth, lateral ones larger than teeth in the middle, teeth in *Salganea incerta* contiguous and subequal; 4) *L2d* with rounded apex and lateral sclerite relatively larger, apex acute and lateral sclerite smaller in *Salganea incerta*.

##### Etymology.

The scientific epithet of this species is derived from the Latin word “*anisodontus*” which refers to the different teeth in the hind margin of the supra-anal plate.

#### Salganea taiwanensis

Roth, 1979

http://species-id.net/wiki/Salganea_taiwanensis

Figs 7–8 , 37–45 , 83–84 , 92–93 , 98–113

Salganea taiwanensis Roth, 1979: 64.
Salganea panesthiodes Princis (nom. nud.), Asahina 1984: 118.
Salganea taiwanensis taiwanensis Asahina, 1988: 261.
Panesthia concinna Feng & Woo, 1990: 214. Syn. n.

##### Description.

**Male.** Body dark reddish brown, darker on caudal segments, or totally black (Fig. 7). Eyes black-brown and ocelli yellowish. Antennae, upper lip, mandible, labial palpi and maxillary palpomeres brown. Legs dark reddish brown, paler on coxae and trochanter. Abdominal sternites reddish brown with the middle of the first and second sternites brown (Fig. 8).

**Figures 37–45.**
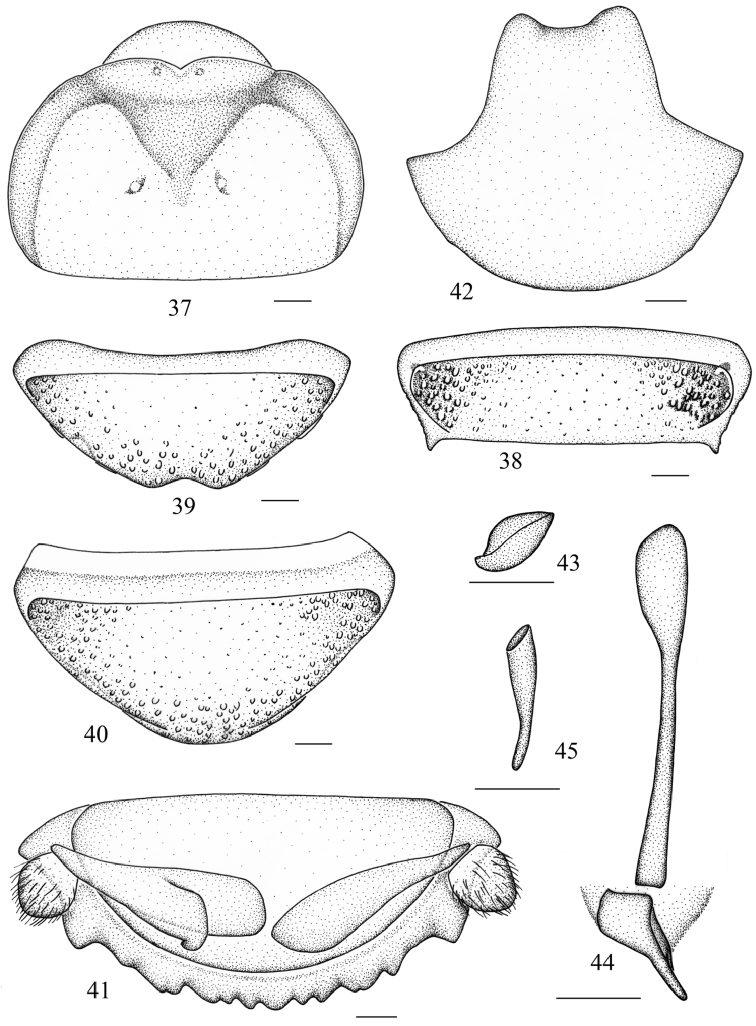
*Salganea taiwanensis* Roth, 1979 **37** vertex and pronotum **38** abdominal tergum 7, dorsal view **39** abdominal sternite 7 of male, ventral view **40** abdominal sternite 7 of female, ventral view **41** supra-anal plate and paraprocts, ventral view **42** subgenital plate, dorsal view **43** left phallomere (*L1*) **44** median phallomere (*L2vm* and *L2d*) **45** right phallomere (*R2*). Scale bars = 1.0 mm (Figs **37–40**), 0.5 mm (Figs **41–45**).

Vertex with few punctations, exposed; face densely punctulated; ocelli round and distinct. Anterior margin of pronotum thickened, with a small mesial V-shaped indentation, a small reflexed protuberance on the each side of the excision; anterior half of pronotum depressed, with sparsely granular surface; posterior half punctured, with 2 oblique mounds armed with 2 tubercles at apex (Fig. 37). Tegmina and wings well developed, extending beyond the abdominal terminal (Fig. 7). In tegmen, the radius with 3 short or 1 relatively long posterior branches; median vein simple or branched (Figs 83–84). Radius in hind wing with apical posterior branch near the middle, which is associated with 2 branches and one of them sometimes branched, some veins fused partially; median vein branched or not; cubitus with 5–6 complete and 6-8 incomplete branches (Figs 92–93). Anterior ventral margin of front femur with 0-4 spines and a small distal spine, hind margin with a large distal spine. Abdominal tergites punctured, and the punctures denser laterally; *T4*–*T7* with holes on the anterolateral corners which are surrounded with fine hairs, the holes on *T4* small or absent; lateral margins of *T7* feebly crenulate, sometimes the irregularities subobsolete and indistinct, caudal angle oblique (Fig. 38). Abdominal sternites densely punctured, hind margin with a mesial concavity (Fig. 39). Supra-anal plate densely punctate, hind margin with 8–13 subequal teeth (10 in most cases), which are broad basally, triangular, or fused together, lateral angles obtuse and about the same size as the largest tooth between them. Cercus conical, very few or no setae dorsally, ventral surface swollen, densely setose (Fig. 41). Anterior margin of subgenital plate slightly concave, lateral margins sunken (Fig. 42).

**Male genitalia.** Genital phallomere *L1* reduced or absent (Fig. 43); *L2d* elongate, apically acute and forked, with a small lateral lobe (Fig. 44); *R2* variably reduced and usually not hook-shaped, or absent (Fig. 45).

**Female.** Essentially similar to male, but larger than male and with hind margin of the seventh sternite rounded (Fig. 40).

##### Measurements.

Male, 3^th^–5^th^ maxillary segments: 0.65–0.75/0.63–0.89/0.86–1.01mm; pronotum: length × width: 4.7–6.2 × 7.7–10.5mm; distance between disc tubercles: 1.8–2.8mm; tegmen: 24.0–27.0mm; body length: 24.0–30.5mm; fore leg: coxae: 2.60–3.19mm, trochanter: 1.61–1.71mm, femur: 2.71–4.07mm, tibia: 1.30–2.33mm, 1^st^–5^th^ tarsus: 0.62–0.67/0.21–0.22/0.19–0.22/0.24–0.32/1.37–1.44mm; mid leg: coxae: 2.76–3.00mm, trochanter: 2.33–2.39mm, femur: 4.35–5.01mm, tibia: 3.81–4.84mm, 1^st^–5^th^ tarsus: 0.80–0.82/0.26–0.28/0.26–0.27/0.28/1.07–1.11mm; hind leg: coxae: 2.44–2.94mm, trochanter: 2.52–2.60mm, femur: 4.71–5.58mm, tibia: 5.67–7.21mm, 1^st^–5^th^ tarsus: 1.06–1.22/0.26–0.35/0.26–0.34/0.28–0.41/1.07–1.33mm; cerci: 0.68–0.79mm.

Female, 3^th^–5^th^ maxillary segments: 0.68–0.85/0.66–0.75/0.59–0.94mm; pronotum: length × width: 4.7–5.8 × 8.0–13.0mm; distance between disc tubercles: 1.8–2.7mm; tegmen: 24.1–27.5mm; body length: 25.0–29.5mm; fore leg: coxae: 3.30–3.78mm, trochanter: 1.99–2.01mm, femur: 3.19–3.41mm, tibia: 2.05–2.06mm, 1^st^–5^th^ tarsus: 0.68–0.78/0.23–0.29/0.22–0.27/0.25–0.29/0.77–1.00mm; mid leg: coxae: 2.85–3.39mm, trochanter: 2.29–2.37mm, femur: 4.37–4.39mm, tibia: 3.80–4.03mm, 1^st^–5^th^ tarsus: 0.79–0.96/0.24–0.30/0.23–0.27/0.28–0.33/1.05–1.14mm; hind leg: coxae: 2.67–3.03mm, trochanter: 2.44–2.45mm, femur: 4.90–5.04mm, tibia: 6.29–6.47mm, 1^st^–5^th^ tarsus: 1.13–1.28/0.28–0.31/0.30–0.32/0.25–0.37/1.11–1.16mm; cerci: 0.61–0.68mm.

##### Material examined.

One male (holotype of *Panesthia concinna* Feng & Woo, 1990), Fujian Prov., Mt. Wuyishan, 10–17 July 1982, coll. Feng Xia; one male, Fujian Prov., Mt. Wuyishan, 15 July 1984, coll. Sizheng Wang; two males and one female, Jiangxi Prov., Mt. Jiulianshan, Gongtang, 30 April 1986, coll. Jianzhong Zheng; four males and one female, Jiangxi Prov., Mt. Jiulianshan, 4 May 1986, coll. Liu Luo; two females, Fujian Prov., 30 June 1982, coll. Fan Jiang; one female, Guangxi Prov., Huaping Nature Preserves, Mt. Tianpingshan, coll. Kun Yang; two females, Guizhou Prov., Ceheng County, 800–950m, 23–27 July 1979, coll. Shaokun Du; one male, Guangdong Prov., Meizhou City, Mt. Wuzhishan, May 2007, coll. Lijun Cai. (SWU)

##### Remarks.

Considering the *Panesthia*-like lateral margin on *T7* and the disappearance of sclerite *R2*, Feng and Woo (1990) treated *Panesthia concinna* (Figs 7–8) as a member of the genus *Panesthia*. But after examining the holotype of *Panesthia concinna* Feng & Woo, 1990, the essentially straight lateral margin of *T7* and the absence of *R2* should be within the intraspecific variation of *Salganea taiwanensis*. Herein we treat *Panesthia concinna* Feng & Woo, 1990 as a synonym of *Salganea taiwanensis* Roth, 1979.

##### Distribution.

China (Jiangxi, Fujian, Guangxi, Guizhou, Guangdong, Taiwan); Japan; Vietnam.

#### Salganea guangxiensis

(Feng & Woo, 1990) comb. n.

http://species-id.net/wiki/Salganea_guangxiensis

Figs 9–10 , 21–24 , 46–49

Panesthia guangxiensis Feng & Woo, 1990: 214.

##### Description.

**Male.** Body dark reddish, with the coloration similar to *Salganea taiwanensis*.

**Figures 46–49:**
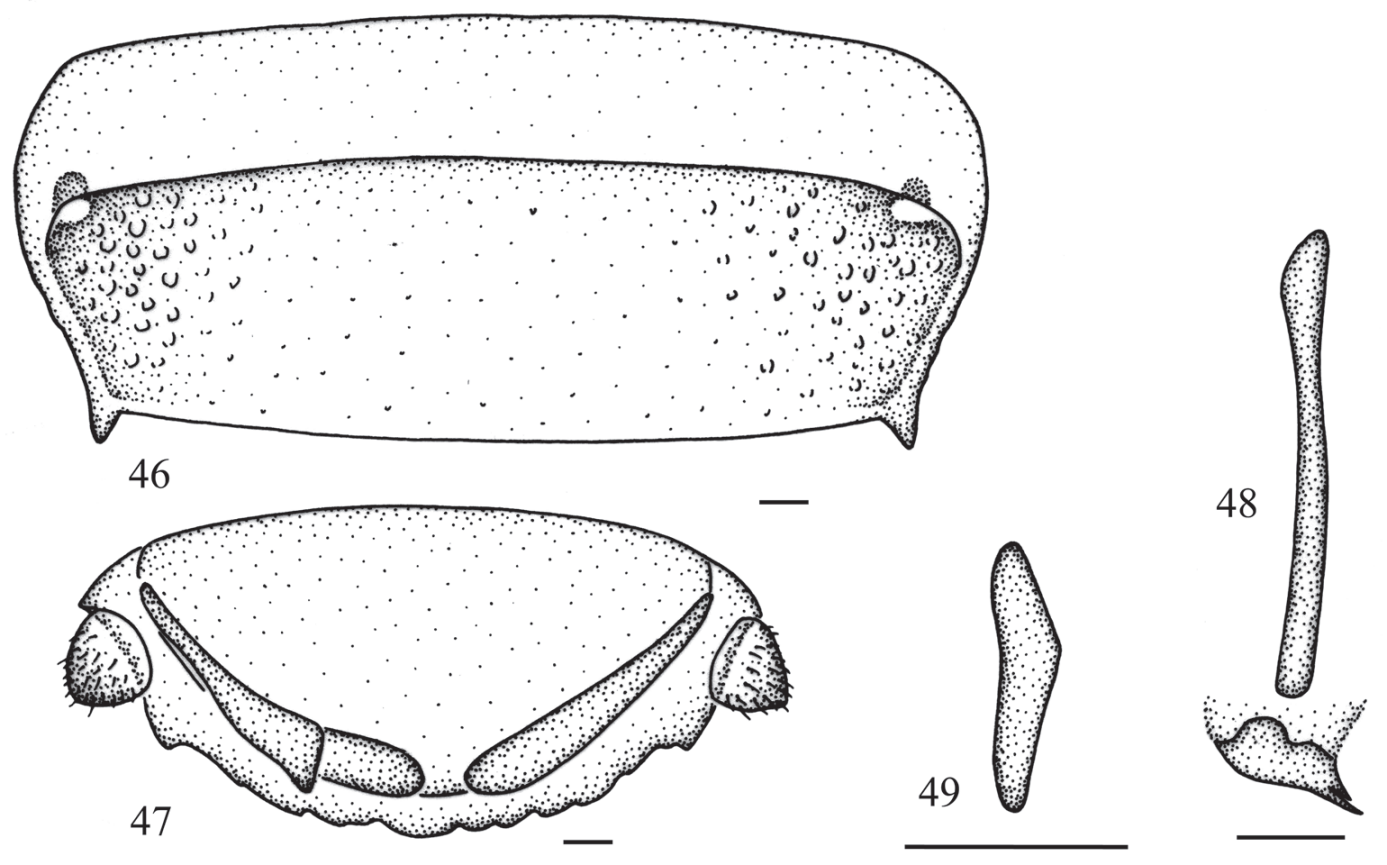
*Salganea guangxiensis* (Feng *et* Woo, 1990) **46** abdominal tergum 7, dorsal view **47** supra-anal plate and paraprocts, ventral view **48** median phallomere (*L2vm* and *L2d*) **49** right phallomere (*R2*). Scale bars = 0.5 mm.

Vertex sparsely punctate, exposed; face densely punctulated; ocelli round and distinct. Anterior margin of pronotum weakly thickened, with a small mesial emargination, a protuberance on the each side of the excision; anterior half of pronotum depressed, the surface sparsely granular; posterior half punctured, with 2 tubercles (Fig. 9). Tegmina and wings mutilated, probably fully developed (Fig. 9). Anterior ventral margin of front femur with 2 spines and a small distal spine, hind margin with a large distal spine. *T4*–*T7* with holes on the anterolateral corners which are surrounded by fine hairs, the holes on *T4* are very small; lateral margins of *T7 Panesthia*-like (Fig. 46). Supra-anal plate densely punctate, hind margin with 10 subequal teeth, obtuse rounded (Fig. 47). The last abdominal sternite and subgenital plate were broken.

**Male genitalia.** Genital phallomere *L1* lost; *L2d* elongate, apically acute and forked, with a lateral lobe (Fig. 48); *R2* reduced, clavate (Fig. 49).

##### Measurements.

Male, 3^th^–5^th^ maxillary segments: 0.73/0.76/1.05mm; pronotum: length × width: 5.1 × 9.5mm; distance between disc tubercles: 2.2mm; body length: 27.4mm; fore leg: coxae: 2.42mm, trochanter: 1.64mm, femur: 3.47mm, tibia: 2.48mm, 1^st^–5^th^ tarsus: 0.70/0.30/0.19/0.26/1.34mm; mid leg: coxae: 3.31mm, trochanter: 2.74mm, femur: 4.33mm, tibia: 3.96mm; hind leg: coxae: 2.57mm, trochanter: 2.72mm, femur: 4.43mm, tibia: 5.92mm; cerci: 0.71mm.

##### Material examined.

One male (holotype of *Panesthia guangxiensis* Feng & Woo, 1990), China: Guangxi Prov., Mt. Jinxiulaoshan, 6 September 1981, collector unknown. (SWU)

##### Remarks.

Although the lateral margin of *T7* is *Panesthia*-like, the characters of the holes in anterolateral corners of *T4*–*T7* associated with setae, and the ridge along with the lateral margin of *S7* are more typical of the genus *Salganea*. Thus, we place this species in genus *Salganea*. It is very similar to *Salganea taiwanensis*, only differing in the rounded teeth in the hind margin of supra-anal plate. To be rigorous, the relationship between this two species requires more specimens to provide an absolute determination.

##### Distribution.

China (Guangxi).

#### Salganea incerta

(Brunner von Wattenwyl, 1893)

http://species-id.net/wiki/Salganea_incerta

Figs 11–14 , 50–60 , 85–86 , 94–95

Panesthia incerta Brunner von Wattenwyl, 1893: 50.
Salganea incerta , Roth 1979: 73, type species by lectotype.

##### Description.

**Male.** Body reddish brown, darker caudally (Fig. 11). Eyes blackish brown; ocelli yellowish. Antennae, upper lip, mandible, labial palpi and maxillary palpomeres reddish brown and only a little paler than body. Legs reddish brown, paler on coxae and trochanter. Abdominal sternites reddish brown with the middle of the former two sternites brown (Fig. 12).

**Figures 50–60.**
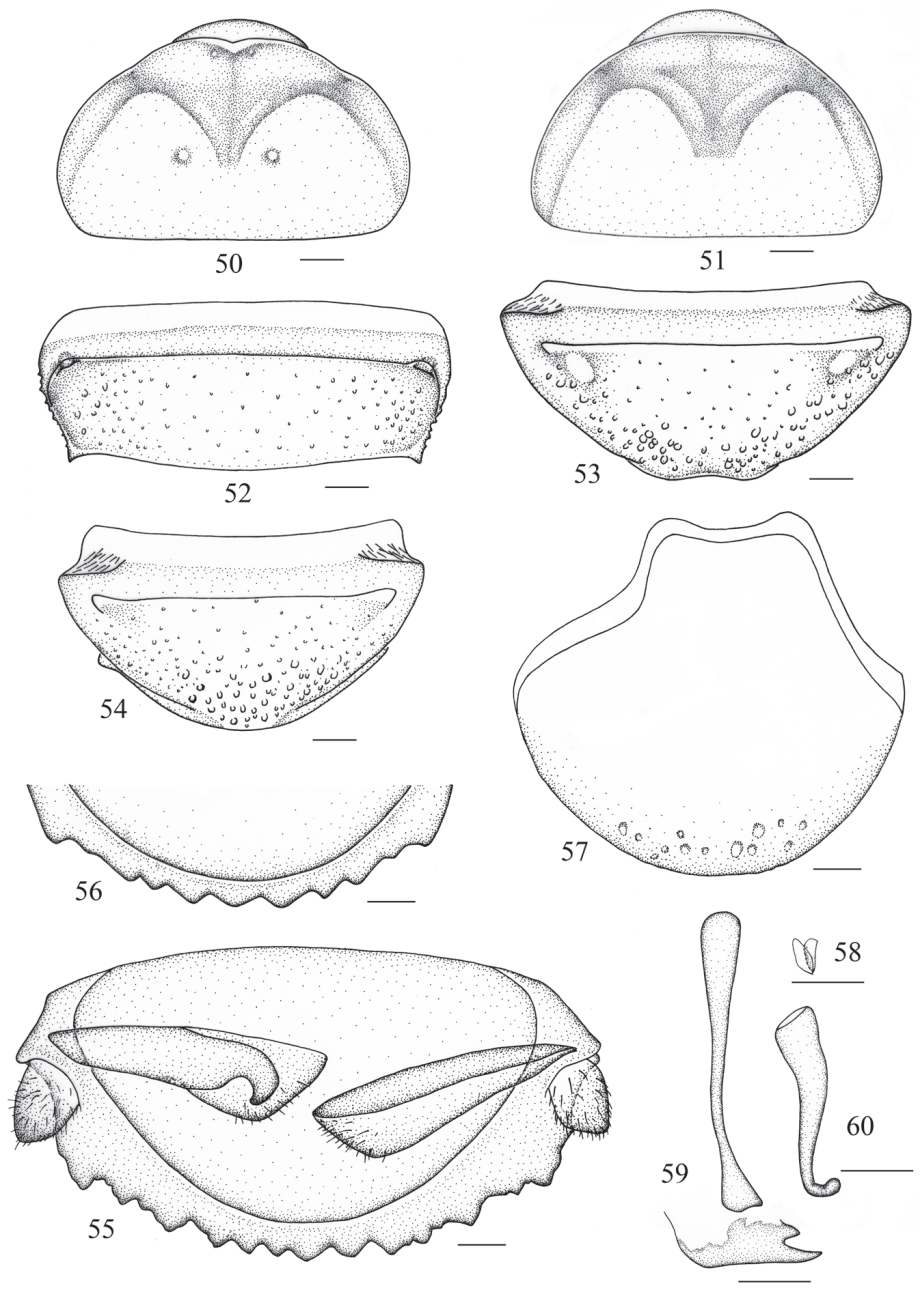
*Salganea incerta* (Brunner von Wattenwyl, 1893) **50** vertex and pronotum, male **51** vertex and pronotum, female **52** abdominal tergum 7, dorsal view **53** abdominal sternite 7 of male, ventral view **54** abdominal sternite 7 of female, ventral view **55** supra-anal plate and paraprocts, ventral view **56** hind margin of supra-anal plate, ventral view **57** subgenital plate, dorsal view **58** left phallomere (*L1*) **59** median phallomere (*L2vm* and *L2d*) **60** right phallomere (*R2*). Scale bars = 1.0 mm (Figs **50–54**), 0.5 mm (Figs **55–60**).

Face sparsely punctate and vertex exposed. Anterior margin of pronotum slightly concave mesially, with a small tubercle on each side of the excision; anterior half slightly depressed, the floor with sparse pustules; few punctations on posterior half, with a pair of small tubercles (Fig. 50). Tegmina and wings extending beyond the end of abdomen, sometimes mutilated (Fig. 11). Radial vein of tegmen with 1 forked posterior branch at base, or with 2 simple posterior branches; median vein branched before the midline (Figs 85–86). Radial vein of hind wing simple and branched apically, or with 3 small branches which terminate in the anterior apical angle; median vein branched; cubitus with 5 complete branches, which may be forked or not, and 7–8 incomplete branches (Figs 94–95). Anteroventral margin of front femur armed with 1 spine or unarmed, with or without a minute distal spine, hind margin with a distal spine. Abdominal tergites 1–6 sparsely punctate; *T7* denser punctulated caudally, lateral margin weakly crenulate, laterocaudal angle slightly produced (Fig. 52); anterolateral corners with small holes in *T6* and *T7*, some minute and indistinct in *T5*. Abdominal sternites punctulate, the last sternite densely punctuated and hind margin indented (Fig. 53). Supra-anal plate densely punctate, hind margin varied with 9–13 triangular teeth, border of teeth smooth or crenulate (Figs 55–56). Cercus conical, with few setae near the border dorsally and ventral surface setose (Fig. 55). Anterior margin of subgenital plate slightly concave, lateral margins slightly curved (Fig. 57).

**Male genitalia.** *L1* reduced, only two small lobes remained (Fig. 58); *L2d* forked apically and apex acute (Fig. 59); *R2* hook-shaped, with weak hook portion and the apex not curved upwards (Fig. 60).

**Female.** Differs from male as follows: anterior margin of pronotum smooth or indented mesially and the tubercles absent or weakly indicated (Fig. 51); hind margin of S7 entire (Fig. 54).

**Nymph.** Body brown, darker caudally (Figs 13–14), the depression of pronotum punctate.

##### Measurements.

Male, 3^th^–5^th^ maxillary segments: 0.36–0.59/0.64–0.78/0.90–0.94mm; pronotum: length × width: 3.6–5.2 × 6.5–8.7mm; distance between disc tubercles: 1.4–2.2mm; tegmen: 24.0–26.5mm; body length: 17.7–26.2mm; fore leg: coxae: 1.53–2.29mm, trochanter: 1.65–1.86mm, femur: 2.81–3.19mm, tibia: 1.37–1.55mm, 1^st^–5^th^ tarsus: 0.34–0.63/0.17–0.20/0.19–0.23/0.24–0.29/1.00–1.18mm; mid leg: coxae: 2.22–2.51mm, trochanter: 2.12–2.58mm, femur: 4.48–4.90mm, tibia: 3.73–3.79mm, 1^st^–5^th^ tarsus: 0.76–0.99/0.22/0.23/0.26–0.30/1.00–1.03mm; hind leg: coxae: 2.02–2.84mm, trochanter: 2.34–2.56mm, femur: 4.43–5.16mm, tibia: 5.43–5.91mm, 1^st^–5^th^ tarsus: 1.02–1.20/0.25–0.32/0.26–0.28/0.30–0.32/1.12–1.17mm; cerci: 0.73–0.89mm.

Female, 3^th^–5^th^ maxillary segments: 0.70–0.74/0.59–0.66/0.96–0.99mm; pronotum: length × width: 4.4–5.8 × 7.6–9.1mm; body length: 20.0–27.0mm; fore leg: coxae: 2.27–2.54mm, trochanter: 1.68–1.93mm, femur: 3.52–3.54mm, tibia: 1.64–2.00mm, 1^st^–5^th^ tarsus: 0.54–0.56/0.16–0.21/0.20–0.26/0.27–0.30/1.10–1.17mm; mid leg: coxae: 2.34–3.02mm, trochanter: 1.63–1.99mm, femur: 4.67–4.75mm, tibia: 4.25–4.46mm, 1^st^–5^th^ tarsus: 1.08–1.11/0.29–0.30/0.25/0.30–0.31/1.01–1.13mm; hind leg: coxae: 2.11–2.51mm, trochanter: 2.01–2.30mm, femur: 5.02–5.60mm, tibia: 6.04–6.19mm, 1^st^–5^th^ tarsus: 1.02–1.17/0.30–0.34/0.27–0.28/0.32–0.33/1.00–1.13mm; cerci: 0.54–0.94mm.

##### Material examined.

Two males, Guangxi Prov., Jinxiu County, Mt. Yangjiaoshan, 25 September 1981, collector unknown; one male and two females, Chongqing, Mt. Simianshan, Dawopu, 11 July 2008, coll. Zongqing Wang; one male and one female, Sichuan Prov., Hongya County, Mt. Wawushan, 30 June 2013, coll. Yang Li and Jinjin Wang; one male, Yunnan Prov., Yingjiang County, 1418m, 24°61'N, 97°62'E, 4–5 June 2008, coll. Weiwei Zhang; two males, Yunnan Prov., Yingjiang County, Xima Township, Menglaihe River 2nd Hydroelectric Power Station, 1470m, 24°78.404'N, 97°67.493'E, 27–29 May 2009, coll. Weiwei Zhang; two males, Yunnan Prov., Yingjiang County, Xima Township, Menglaihe 2nd Hydroelectric Power Station, 1470m, 6–9 June 2008, coll. Weiwei Zhang; two males, Yunnan Prov., Yingjiang Country, Taiping Town, Longpen Village, 30 May-9 June 2009, coll. Weiwei Zhang; one male and one female, Hainan Prov., Mt. Diaoluoshan, 18°43.462'N, 108°52.105'E, 4 May 2013, coll. Yan Shi and Shunhua Gui; one male and one female, China, 4 May 1980, coll. Qiaosheng Yuan; two males, two females and 3 nymphs, Chongqing, Mt. Simianshan, 2 October 2013, coll. Hao Xu and Jianyue Qiu. (SWU)

##### Distribution.

China (Guangxi, Chongqing, Sichuan, Yunnan, Hainan); India; Myanmar; Thailand.

#### Salganea raggei

Roth, 1979

http://species-id.net/wiki/Salganea_raggei

Figs 15–18 , 61–68 , 87–88 , 96–97

Salganea raggei Roth, 1979: 30.

##### Description.

**Male.** Body reddish brown, darker caudally, or black (Fig. 15). Eyes black and ocelli yellowish. Antennae, upper lip, mandible, labial palpi and maxillary palpomeres reddish brown. Legs brown, reddish on coxae, trochanter and anterior half of femora. Abdominal sternites reddish brown with the middle of the former several sternites brown (Fig. 16).

**Figures 61–68.**
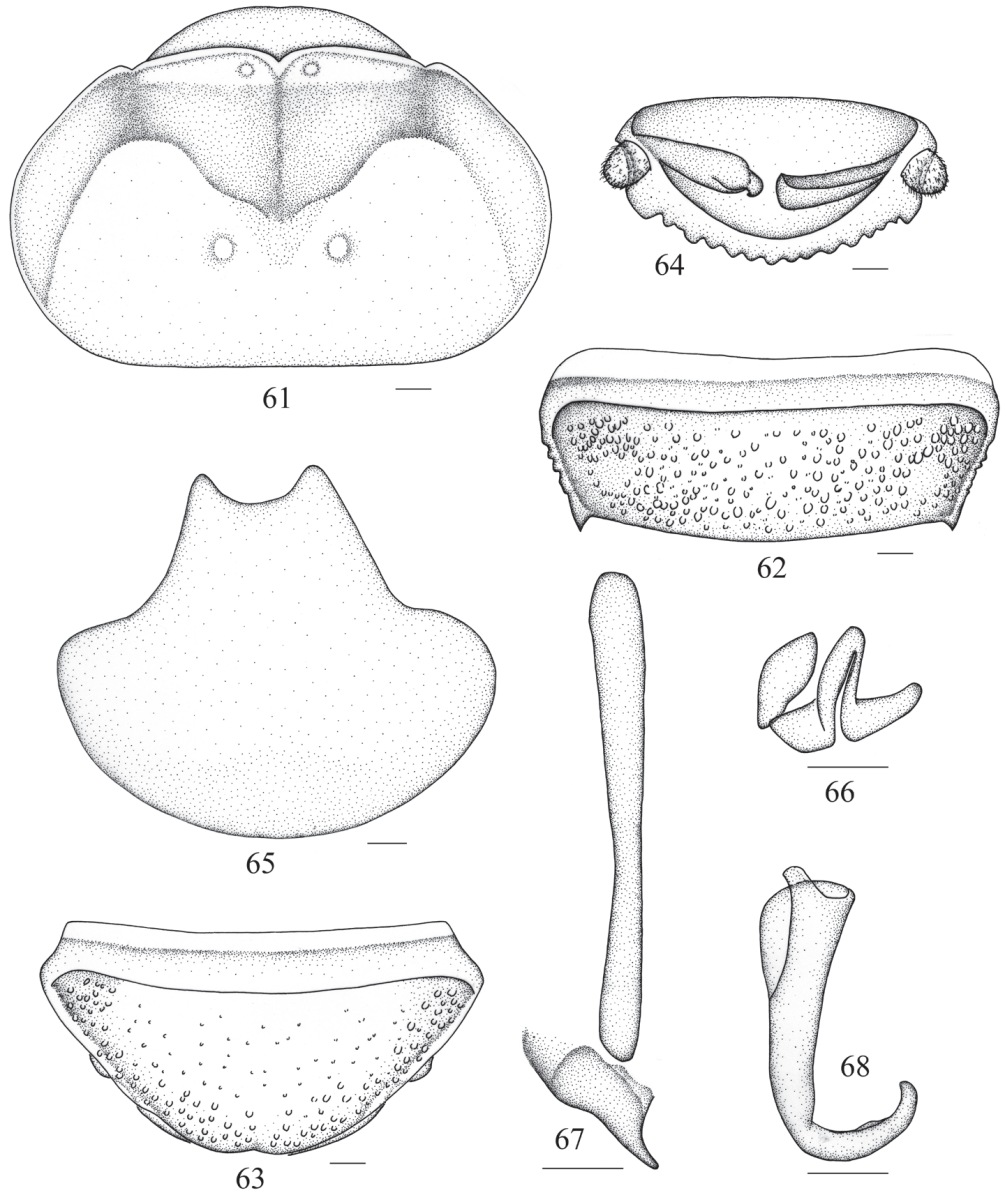
*Salganea raggei* Roth, 1979 **61** vertex and pronotum **62** abdominal tergum 7, dorsal view **63** abdominal sternite 7, ventral view **64** supra-anal plate and paraprocts, ventral view **65** subgenital plate, dorsal view **66** left phallomere (*L1*) **67** median phallomere (*L2vm* and *L2d*) **68** right phallomere (*R2*). Scale bars = 1.0 mm (Figs **61–64**), 0.5 mm (Figs **65–68**).

Face punctulated and vertex exposed. Ocelli small and round. Anterior margin of pronotum with a V-shaped emargination in the midline, a small recurved tubercle behind the margin on each side of the excision; anterior half depressed, the floor densely granular, lateral area punctate; region behind grooves convex and densely punctate, with one or two pairs of relatively large tubercles on the mounds symmetrically (Fig. 61). Tegmina and wings well developed, sometimes mutilated and remaining an uneven base (Fig. 15). Radius of tegmen with or without 1 long posterior branch which is forked; median vein branched (Figs 87–88). Radial vein of hind wing with one or more branches near the apex, some branches reforked, the posterior branch branched or not; median vein with two branches or simple; cubitus with 7–8 complete and 8–10 incomplete branches (Figs 96–97). Anterior ventral margin of fore femur equipped with 1–3 spines, and with or without a minute distal spine, posterior margin with a large distal spine. Abdominal tergites punctated, the punctures denser laterally and dorsoposteriorly, anterolateral corners usually without holes, rarely with small holes on *T6* and *T7* only; lateral angle of *T6* produced into a rounded and oblique spine; lateral margin of *T7* with 5–6 obtuse teeth, laterocaudal angle stout and subacute (Fig. 62). Abdominal sternites densely punctured, *S7* with a depression on the caudal margin (Fig. 63), subgenital plate more or less exposed. Supra-anal plate densely covered with coarse punctations, sometimes with minute hairs; hind margin with 8-16 subequal teeth, whose apex obtuse; some teeth contiguous. Cerci bulbous, dorsal surface hairless and ventral surface setose (Fig. 64). Subgenital plate with anterior margin concave, lateral margin with a mesal indentation (Fig. 65).

**Male genitalia.** *L1* developed but slightly sclerotized (Fig. 66); *L2d* not bifurcated, elongate and tapering towards the apex, mostly with a weak concavity in hind margin (Fig. 67); *R2* well developed, hook-shaped, apex subacute and curved upwards (Fig. 68).

**Female.** Differs only slightly from male in partial specimens as follows: anterior margin of pronotum slightly excised, and the tubercles indistinct behind the indentation.

**Nymph.** Similar to adult, but body yellowish-brown, anterior margin of the pronotum without excision and protuberance, the tubercles on the floor absent; depression of anterior half densely punctuate; meso- and metanotum with produced laterocaudal angle, the protrusion dark or black (Figs 17–18).

##### Measurements.

Male, 3^th^–5^th^ maxillary segments: 0.86–0.90/0.88–1.29/1.15–1.67mm; pronotum: length × width: 6.3–10.1 × 10.0–16.0 mm; distance between anterior disc tubercles: 5.4–7.7 mm; distance between posterior disc tubercles: 2.4–4.7mm; tegmen: 38.0–51.0mm; body length: 29.0–49.0 mm; fore leg: coxae: 4.26–4.93mm, trochanter: 2.67–3.17mm, femur: 4.98–5.65mm, tibia: 2.65–3.23mm, 1^st^–5^th^ tarsus: 0.88–0.99/0.27–0.36/0.30–0.35/0.36–0.55/1.51–1.65mm; mid leg: coxae: 4.35–5.04mm, trochanter: 2.87–4.25mm, femur: 6.39–6.84mm, tibia: 5.47–5.87mm, 1^st^–5^th^ tarsus: 1.18–1.47/0.31–0.40/0.31–0.43/0.40–0.58/1.34–1.98mm; hind leg: coxae: 3.81–5.02mm, trochanter: 3.13–4.39mm, femur: 7.26–7.44mm, tibia: 7.54–9.98mm, 1^st^–5^th^ tarsus: 1.42–1.47/0.44–0.51/0.37–0.45/0.43–0.66/1.60–2.10mm; cerci: 1.00–1.22mm.

Female, 3^th^–5^th^ maxillary segments: 0.95–1.14/0.73–0.98/1.12–1.37mm; pronotum: length × width: 7.0–12.0 × 11.0–16.5 mm; distance between posterior disc tubercles: 2.5–4.7 mm; tegmen: 33.0–50.0mm; body length: 30.5–49.0 mm; fore leg: coxae: 2.29–5.01mm, trochanter: 2.97–3.33mm, femur: 4.62–4.91mm, tibia: 2.39–2.67mm, 1^st^–5^th^ tarsus: 0.89–1.11/0.31–0.32/0.30–0.32/0.31–0.42/1.57–1.85mm; mid leg: coxae: 3.99–4.43mm, trochanter: 4.08–4.29mm, femur: 6.58–6.69mm, tibia: 5.54–5.85mm, 1^st^–5^th^ tarsus: 1.10–1.24/0.29–0.38/0.27–0.39/0.42–0.45/1.30–1.50mm; hind leg: coxae: 3.94–4.56mm, trochanter: 3.66–4.27mm, femur: 6.54–7.44mm, tibia: 7.91–9.35mm, 1^st^–5^th^ tarsus: 1.22–1.64/0.32–0.40/0.32–0.38/0.38–0.52/1.15–2.19mm; cerci: 0.97–1.20mm.

##### Material examined.

One male and two females, Yunnan Prov., Damenglong Town, 650m, 13 April 1958, coll. Chunpei Hong; one male and one female, Yunnan Prov., Damenglong Town, 650m, 16 March 1958, coll. Zhizi Chen; one male, one female and one nymph, Yunnan Prov., Damenglong Town, 650m, 18 April 1958, coll. Fuji Pu; three males, Yunnan Prov., Yingjiang County, Tongbiguan Township, 1418m, 24°61'N, 97°62'E, 4–5 June 2008, coll. Weiwei Zhang; one male, Xizang Prov., Motuo County, 1300m, 10 September 1979, coll. Gentao Jin and Jianyi Wu; one female, Xizang Prov., Motuo County, Gedang Township, 2080m, 15-18 April 1980, coll. Gentao Jin and Jianyi Wu; two males, Hainan Prov., Mt. Jianfengling, 22 February 1982, collector unknown; one female, Hainan Prov., Mt. Jianfengling, 10 May 1964, coll. Sikong Liu; two males and one nymph, Hainan Prov., Mt. Jianfengling, 4 May 2013, coll. Yan Shi and Shunhua Gui; one male, Hainan Prov., Wuzhishan city, shuiman Township, 740m, 18°51'N, 109°40', 28–30 June 2008, coll. Weiwei Zhang; one male, Hainan Prov., Mt. Jianfengling, 15 June 1983, collector unknown; one male, Hainan Prov. Mt. Jianfengling, Tianchi, 25 April 1981, coll. Shaoying Liang. (SWU).

##### Distribution.

China (Yunnan, Xizang, Hainan, Taiwan); Bhutan; India; Laos; Nepal; Vietnam; Sikkim; Thailand.

#### Salganea flexibilis sp. n.

http://zoobank.org/F372D347-53DE-4495-8E4A-252371FA93B1

http://species-id.net/wiki/Salganea_flexibilis

Figs 19–20 , 69–76

##### Description.

**Male.** Body black (Fig. 19). Head black, dark reddish brown between eyes, ocelli and eyes with yellowish border. Antennae, upper lip, mandible, labial palpi and maxillary palpomeres dark brown. Legs black, coxae, trochanter and the former half of femora yellowish brown. Abdominal sternites black with the middle of the former five segments reddish brown, cercus reddish brown (Fig. 20).

**Figures 69–76.**
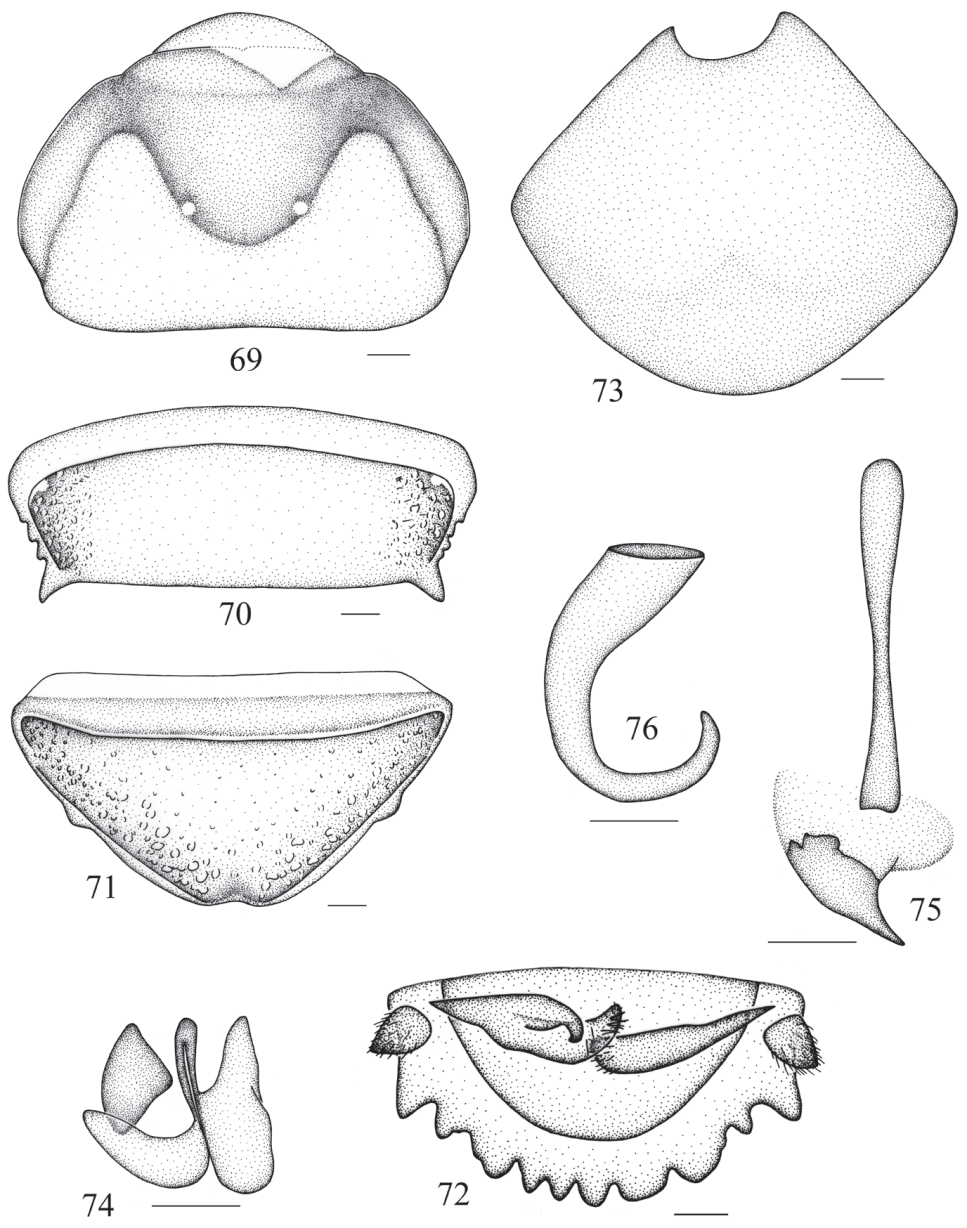
*Salganea flexibilis* sp. n. **69** vertex and pronotum **70** abdominal tergum 7, dorsal view **71** abdominal sternite 7, ventral view **72** supra-anal plate and paraprocts, ventral view **73** subgenital plate, dorsal view **74** left phallomere (*L1*) **75** median phallomere (*L2vm* and *L2d*) **76** right phallomere (*R2*). Scale bars = 1.0 mm (Figs **69–72**), 0.5 mm (Figs **73–76**).

Vertex exposed, punctured; face densely punctured; ocelli circular and distinct. Pronotum convex (anterior margin damaged, it is probably a developmental error), anterior and lateral area depressed, densely granular and equally distributed; posterior half with a distinct tubercle on each side of the midline, the floor densely punctured (Fig. 69). Tegmina and wings mutilated, but probably fully developed (Fig. 19). Anterior ventral margin of front femur with 2 spines, and a minute distal spine, posterior margin with a spine. Centre region of abdominal tergites sparsely punctured, more and larger laterally, and with dense setae; lateral angle of *T6* produced; *T7* hirsute, with large disc pits densely; round holes only present in the anterolateral corners of *T6* and *T7*; lateral margin of *T7* with 3 subacute and distinct teeth, sometimes also with subobsolete papulas, caudal angle produced into a strong and oblique spine (Fig. 70). Abdominal sternites densely punctured; *S7* densely covered with hairs, hind margin emarginated (Fig. 71), subgenital plate slightly exposed. Supra-anal plate convex, hirsute, with large disc pits densely; middle of hind margin with 8 unequal teeth, deflexed, which are triangular or apically truncate; caudal angles tapering, and same length as the largest tooth between them. Cerci conical, dorsal and ventral surfaces densely setose (Fig. 72). Anterior margin of subgenital plate depressed, anterolateral corners subacute, lateral margins straight and not concave (Fig. 73).

**Male genitalia.** *L1* well developed (Fig. 74); *L2d* not bifurcated with acute apex (Fig. 75); *R2* hook-shaped (Fig. 76).

**Female.** Unknown.

**Nymph.** Unknown.

##### Measurements.

Male, 3^th^–5^th^ maxillary segments: 0.90/0.89/0.99mm; pronotum: length × width: 6.9 × 11.5mm; distance between disc tubercles: 2.8mm; body length: 32.2mm; fore leg: coxae: 2.99mm, trochanter: 2.54mm, femur: 4.04mm, tibia: 2.00mm, 1^st^–5^th^ tarsus: 0.90/0.32/0.32/0.38/1.36mm; mid leg: coxae: 3.50mm, trochanter: 2.88mm, femur: 5.76mm, tibia: 4.34mm, 1^st^–5^th^ tarsus: 1.09/0.33/0.34/0.42/1.30mm; hind leg: coxae: 3.32mm, trochanter: 3.36mm, femur: 6.77mm, tibia: 6.59mm, 1^st^–5^th^ tarsus: 1.27/0.42/0.38/0.45/1.46mm; cerci: 0.93mm.

##### Material examined.

*Holotype*, male, China: Yunnan Prov., Nujiang State, Gongshan County, Dulongjiang Township, Kongmu Village, 1391m, 27°44.79'N, 98°20.19'E, 25 May 2013, coll. Hao Xu and Jianyue Qiu (SWU).

##### Remarks.

Owing to *L2d* not being bifurcated, this species should be placed under the *Salganea raggei* species group. It is superficially similar to *Salganea aperturifera*, but can be differentiated by the following characteristics: 1) posterior half of pronotum with 1 pair of tubercles, 2 pairs in *Salganea aperturifera*; 2) abdominal tergites 6 and 7 with holes in the anterolateral corners, *T3*–*T7* with holes in *Salganea aperturifera*; 3) supra-anal plate convex, hind margin with 8 deflexed and unequal teeth, the teeth but in *Salganea aperturifera* the number of teeth is 8–10, which are subequal and undeflexed.

##### Etymology.

The specific epithet “*flexibilis*” is derived from Latin, which means that the teeth on the hind margin of the supra-anal plate are deflexed.

## Discussion

**Figures 77–79.**
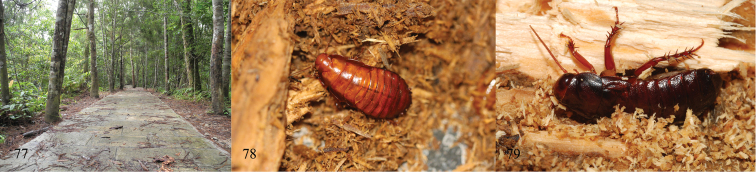
**77** ecotope of Mountain Diaoluoshan, Hainan Province (Photographs by Keliang Wu) **78** nymph of *Salganea quinquedentata* sp. n. **79** *Salganea incerta* (Brunner von Wattenwyl, 1893), in Mountain Simianshan, Chongqing, 2 October 2013 (Photographs by Jianyue Qiu).

The members of *Salganea* are known to be burrowers in rotten logs (Figs 78–79), with a hard, rigid, pitted exoskeletion and a thick, scoop-shaped pronotum (Bell et al. 2007). Some macropterous ones shed their wings and only keep the basal region of tegmina and wings intact (Roth 1979; Maekawa et al. 1999b; Bell et al. 2007), *i.e.*, *Salganea quinquedentata* sp. n., *Salganea flexibilis* sp. n. and *Salganea anisodonta* sp. n. as described in this paper, which are all collected by chopping logs and not by light trapping in tropical forests (Fig. 77). Most of them have shed their tegmina and wings and only one specimen of *Salganea quinquedentata* sp. n. kept these intact; the reason of shedding is maybe the combination of scraping them against gallery walls and the chewing action of conspecifics (Maekawa et al. 2008). The nymphs of *Salganea quinquedentata* sp. n. are not active and slowly crawl on the scraps of wood; but if frightened by sound and light, they will flee and hide below the scraps (Wang X.D., pers. obser.).

**Figures 80–97.**
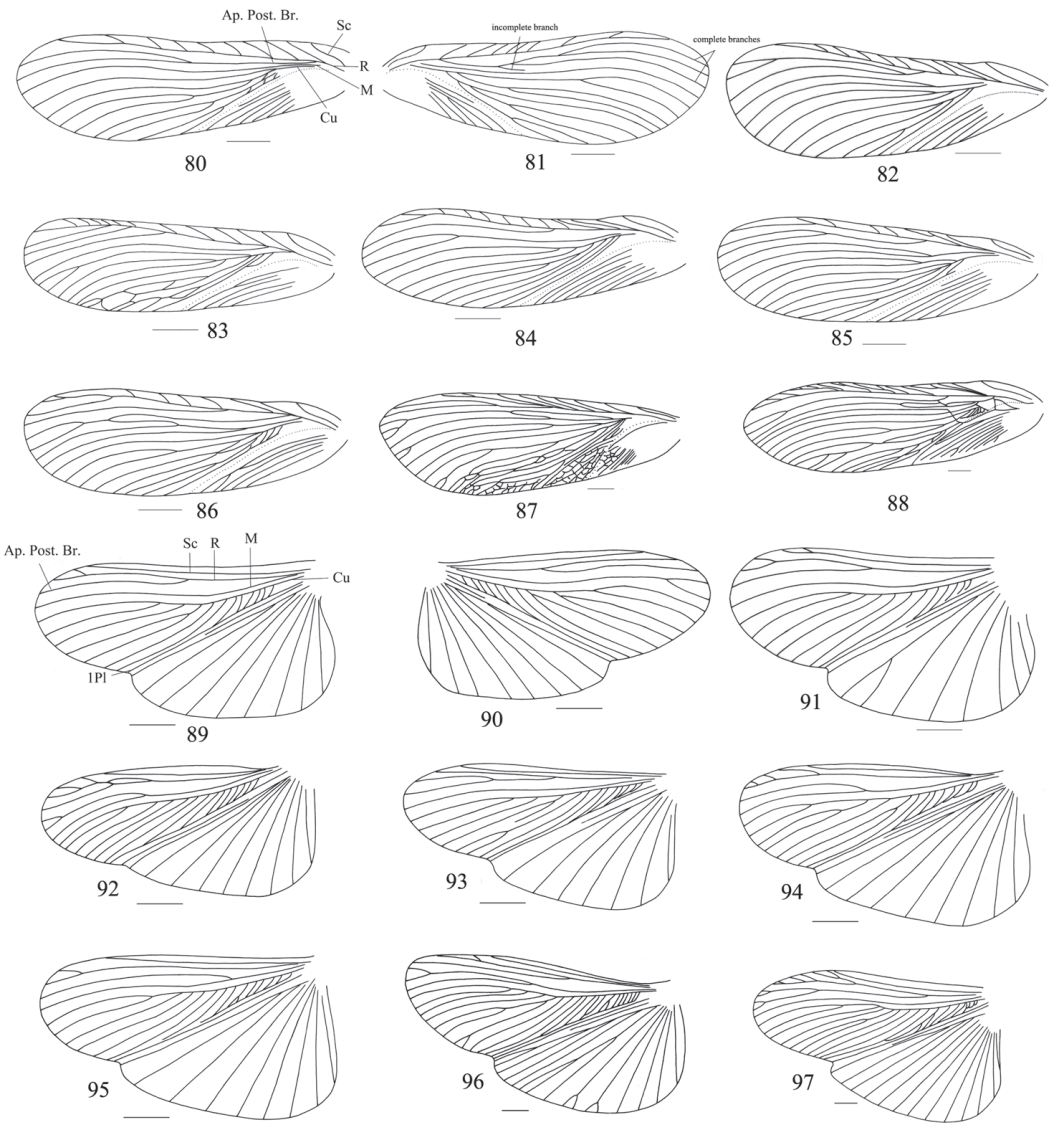
**80–88** tegmina: **80–81** left and right tegmina of one specimen (*Salganea quinquedentata* sp. n.), dorsal view **82** *Salganea anisodonta* sp. n. **83–84** *Salganea taiwanensis* Roth, 1979 **85–86** *Salganea incerta* (Brunner von Wattenwyl, 1893) **87–88** *Salganea raggei* Roth, 1979 **89–97** wings: **89–90** left and right wings of one specimen (*Salganea quinquedentata* sp. n.), dorsal view **91** *Salganea anisodonta* sp. n. **92–93** *Salganea taiwanensis* Roth, 1979 **94–95** *Salganea incerta* (Brunner von Wattenwyl, 1893) **96–97** *Salganea raggei* Roth, 1979. Scale bars = 4.0 mm.

**Figures 98–113.**
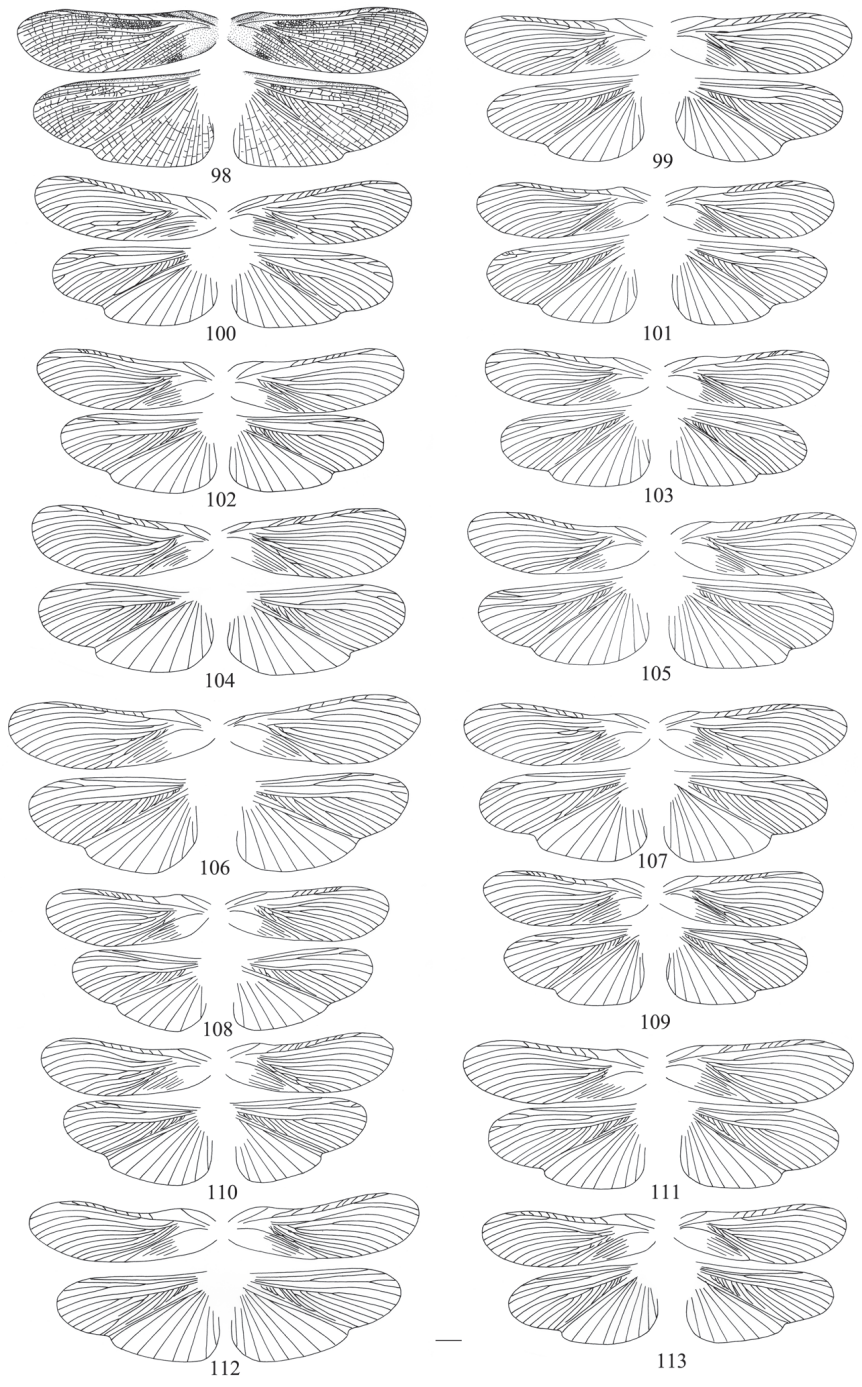
four wings of *Salganea taiwanensis* Roth, 1979 of one specimen, dorsal view **98** wings with cross-veins **99–113** cross-veins of wings omitted. Scale bars = 4.0 mm.

Owing to this unique behavior of shedding their tegmina and wings, venation as an important taxonomic character has not been used widely in the classification of Panesthiinae. But a large number of species of Panesthiinae have fully developed tegmina and wings (Roth 1979). Moreover, Rehn (1951) considered that wings of this taxon had a characteristic venation which differed from other families. But after strict examination we find that the veins of the tegmina and wings are unstable. First, we find the venation of the left and right tegmina and wings of one specimen are variable. As in *Salganea quinquedentata* sp. n., the apical posterior radial vein is branched in the left tegmen (Fig. 80), but has 2 complete and 1 incomplete branches in the right one (Fig. 81); the cubitus has variable branches. The radial vein of the wing bifurcated near the apex, and the median vein is unbranched in the left wing (Fig. 89), but in the right one, the radius is not bifurcated apically, and the median vein branched at the apex (Fig. 90). Second, the intraspecific venation is also variable, as in *Salganea taiwanensis* (Figs 83–84, 92–93, 98–113); venations of all left tegmina illustrated here are significantly different, mainly in the numbers of each vein (Figs 83–84, 98–113). But the total number of veins at the margin appears relatively stable, as in *Salganea taiwanensis*, the CV_total_ of tegmina is 11.19 (Table 1) and CV_total_ of hindwings is 11.11 (Table 2), which are almost same as in the archaic species. The CV of the total number of veins in all living species were under 5.00 (Vršanský 2000). There are mainly 4 kinds of deformities of veins presented in the drawings (*Salganea taiwanensis* of 16 specimens, Figs 98–113), which are expressed as mutual fusion of veins (*Cu*–*Cu*, Figs 99–100, 104, 107–113), lost of a branch (*Cu*, Figs 106–107, 109–110, 112), fusion of vein to another vein (*Cu*–*Cu*, Figs 99–100, 102–108, 110, 111, 113), or as veins with unfinished growth (Fig. 98) in both right and left tegmina. In hindwings, there are deformities expressed as fusion of radial veins (Figs 100–101, 109–110). Mass insects deformities expressed as fusion of wing veins most probably represent heritable mutations (Vršanský 2005). At the same time he mentioned that enhanced environmental stress might have caused the occurrence of mass mutations. Vršanský (2000) also made the conclusion that the variability of the venation of Blattaria species decrease from the archaic to more recent group. So we speculate that the deformities of *Salganea taiwanensis* listed above might be the result of adaptation to the environment, that is, their ancestors may be able fliers but now they have no chance to fly or lack ability of flight before entering the log. However, interspecific venation is relatively stable especially in hindwings (Figs 89–97). The subcosta is simple and long; the radial vein does not have more than five branches; the median vein is with one or without a branch on the anterior part.

**Table 1.** Tegmen venation variability of *Salganea taiwanensis* for 16 specimens The second line numbers are data of left and right tegmina separately. Post. + M – posterior branch of radius + media; Total – total number of veins without radius and anal veins; CV – coefficient of variation. (Liang et al. 2012)

	Sc	R	Post. + M	Cu	Total
**Min**	1 1;1	7 6;7	2 2;2	7 7;8	11 11;11
**Max**	1 1;1	14 12;14	6 5;6	12 12;11	17 17;17
**Median**	1 1;1	9 9;9	3 3.5;3	9 9;9	13 13;13
**Mode**	1 1;1	9 8;9	3 4;3	9 9;8	13 13;14
**Average**	1 1;1	9.563 9.313;9.563	3.344 3.313;3.344	9.063 8.938;9.188	13.406 13.438;13.375
**Deviation**	0 0;0	1.590 1.815;1.590	1.066 1.250;1.066	1.243 1.167;1.243	1.500 1.548;1.500
**CV in %**	0 0;0	16.631 19.495;16.631	31.877 26.224;37.736	13.713 14.994;12.705	11.186 11.519;11.215

**Table 2.** Hindwing venation variability of *Salganea taiwanensis* for 16 specimens. The second line numbers are data of left and right tegmina separately. Total – total number of veins without anal veins; CV – coefficient of variation. (Liang et al. 2012)

	Sc	R	M	Cu	Total
**Min**	1 1;1	1 2;2	1 1;1	4 5;4	10 10;10
**Max**	1 1;1	7 5;7	3 3;2	8 8;8	15 15;15
**Median**	1 1;1	3 3.5;3	1 1;1	6 6.5;6	12 12;12
**Mode**	1 1;1	3 3;3	1 1;1	6 7;6	11 12;11
**Average**	1 1;1	3.625 3.625;3.625	1.188 1.188;1.188	6.406 6.438;6.375	12.188 12.188;12.188
**Deviation**	0 0;0	1.157 0.885;1.408	0.471 0.544;0.403	0.837 0.814;0.885	1.355 1.274;1.471
**CV in %**	0 0;0	31.918 24.415;38.850	39.657 45.803;33.946	13.066 12.644;13.883	11.114 10.473;12.066

Overall, we find venation to be of little value as a specific character; but the venation variation is more stable and distinct at higher taxonomic levels. Additional investigation will be required to search for more stable wing characteristics to support our view.

## Supplementary Material

XML Treatment for Salganea

XML Treatment for Salganea quinquedentata

XML Treatment for Salganea anisodonta

XML Treatment for Salganea taiwanensis

XML Treatment for Salganea guangxiensis

XML Treatment for Salganea incerta

XML Treatment for Salganea raggei

XML Treatment for Salganea flexibilis
